# Associations Between Histo-blood Group Antigen Status in Mother-Infant Dyads and Infant Oral Rotavirus Vaccine Immunogenicity in Rural Zimbabwe

**DOI:** 10.1093/infdis/jiae456

**Published:** 2024-10-01

**Authors:** Joshua Pun, Ceri Evans, Bernard Chasekwa, James A Church, Ethan Gough, Kuda Mutasa, Sandra Rukobo, Margaret Govha, Patience Mushayanembwa, Florence D Majo, Naume V Tavengwa, Jean H Humphrey, Beth D Kirkpatrick, Margaret Kosek, Robert Ntozini, Andrew J Prendergast

**Affiliations:** Centre for Genomics and Child Health, Blizard Institute, Queen Mary University of London, United Kingdom; Zvitambo Institute for Maternal and Child Health Research, Harare, Zimbabwe; Department of Clinical Infection, Microbiology and Immunology, University of Liverpool, United Kingdom; Zvitambo Institute for Maternal and Child Health Research, Harare, Zimbabwe; Centre for Genomics and Child Health, Blizard Institute, Queen Mary University of London, United Kingdom; Zvitambo Institute for Maternal and Child Health Research, Harare, Zimbabwe; Johns Hopkins Bloomberg School of Public Health, Johns Hopkins University, Baltimore, Maryland; Zvitambo Institute for Maternal and Child Health Research, Harare, Zimbabwe; Zvitambo Institute for Maternal and Child Health Research, Harare, Zimbabwe; Zvitambo Institute for Maternal and Child Health Research, Harare, Zimbabwe; Zvitambo Institute for Maternal and Child Health Research, Harare, Zimbabwe; Zvitambo Institute for Maternal and Child Health Research, Harare, Zimbabwe; Zvitambo Institute for Maternal and Child Health Research, Harare, Zimbabwe; Zvitambo Institute for Maternal and Child Health Research, Harare, Zimbabwe; Johns Hopkins Bloomberg School of Public Health, Johns Hopkins University, Baltimore, Maryland; Vaccine Testing Center, Department of Microbiology and Molecular Genetics, College of Medicine, University of Vermont, Burlington; Division of Infectious Diseases and International Health, University of Virginia, Charlottesville; Zvitambo Institute for Maternal and Child Health Research, Harare, Zimbabwe; Centre for Genomics and Child Health, Blizard Institute, Queen Mary University of London, United Kingdom; Zvitambo Institute for Maternal and Child Health Research, Harare, Zimbabwe

**Keywords:** histo-blood group antigens, immunogenicity, oral rotavirus vaccine, secretor status, seroconversion

## Abstract

**Background:**

Histo-blood group antigen (HBGA) phenotypes may contribute to poor oral rotavirus vaccine (RVV) immunogenicity, since rotavirus binds intestinal epithelial HBGA glycans, while maternal HBGA status shapes breastmilk composition, which influences the composition of the infant microbiome. We investigated associations between maternal/infant HBGA phenotypes and RVV immunogenicity in rural Zimbabwe.

**Methods:**

We undertook salivary FUT2/FUT3 phenotyping in mother-infant pairs. Serum anti-rotavirus immunoglobulin A was measured by enzyme-linked immunosorbent assay. We explored adjusted associations between FUT2/FUT3 status and RVV seroconversion (primary outcome, n = 322) and seropositivity and geometric mean titer (secondary outcomes, n = 776).

**Results:**

Infants of FUT2- or FUT3-positive women were less likely to seroconvert post-RVV than infants of FUT2- or FUT3-negative women (FUT2 positive [20.1%] vs FUT2 negative [27.5%]: adjusted relative risk [aRR], 0.47; 95% CI, .26–.82; *P* = .008; FUT3 positive [18.1%] vs FUT3 negative [30.0%]: aRR, 0.45; 95% CI, .25–.78; *P* = .005). When compared with FUT2-positive infants with FUT2-positive mothers, FUT2-positive infants with FUT2-negative mothers were twice as likely to seroconvert (36.8% vs 21.9%; aRR, 2.12; 95% CI, 1.23–3.63; *P* = .006). When compared with FUT3-positive infants with FUT3-positive mothers, FUT3-positive infants with FUT3-negative mothers were 3 times as likely to seroconvert (48.3% vs 18.2%; aRR, 2.99; 95% CI, 1.82–4.90; *P* < .001).

**Conclusions:**

Maternal and infant FUT2 and FUT3 status influences infant RVV immunogenicity.

## Teaser

In an exploration of associations between maternal/infant histo-blood group antigen phenotypes and infant rotavirus vaccine immunogenicity, we show that maternal/infant secretor and Lewis statuses influenced infant immune responses to rotavirus vaccine.

Rotavirus infection is a major contributor to life-threatening diarrhea in children <5 years of age [1, 2]. Since the introduction of 2 live-attenuated oral rotavirus vaccines (RVVs) in 2006, mortality due to rotavirus diarrhea has significantly decreased [3]; however, there were still approximately 150 000 deaths in 2019, predominately in low-income countries [1, 4, 5]. Multiple studies have established that RVV is less efficacious in low-income countries, with a reported efficacy of only 56% as opposed to 85% to 98% in high- and middle-income countries [6–8]. This “efficacy gap” is a major public health concern as it limits the full potential of RVV where it could have the greatest benefits. Several factors have been proposed to identify those that compromise the immunogenicity of RVV, such as greater rotavirus serotype diversity, maternal transplacental and breast milk antibody interference, vaccine formulation, and infant malnutrition [9]. Yet, our understanding of the discrepancies in RVV performance between low- and high-income countries remains poor.

On a population level, genetic factors may influence geographic variation in oral vaccine responses. Histo-blood group antigens (HBGAs) are complex carbohydrates ubiquitously expressed on red blood cells and mucosal surfaces such as the gut, which can also be found in saliva and other exocrine secretions [10]. Their phenotype is determined by fucosyltransferase (FUT) genes, which encode FUT enzymes required for the surface expression and secretion of ABH and Lewis glycans. Individuals with at least 1 functional copy of the *FUT2* gene are characterized as secretors (FUT2 positive) who possess the ability to express or secrete HBGA [10], while individuals homozygous for nonfunctional FUT2 alleles are characterized as nonsecretors (FUT2 negative). Individuals with at least 1 functional copy of the *FUT3* gene are characterized as Lewis positive (FUT3 positive), while individuals homozygous for nonfunctional FUT3 alleles are characterized as Lewis negative (FUT3 negative).

Several strands of evidence provide a rationale for the potential relationship between HBGA status and RVV performance. First, in vitro experiments have demonstrated that rotavirus binds to HBGA glycans through the VP8* domain of protein VP4 in a genotype-specific manner [11–13]. This is consistent with the finding that nonsecretors possess a degree of innate protection against several enteric infections, including the most common rotavirus strain, the P[8] genotype [14–16], from which 2 licensed RVVs are derived [17]. Nonsecretors may therefore have a reduced capacity to bind these RVVs in the gut epithelium. Second, alterations in the fucosylation of the infant intestinal mucosa may affect the maturation of the gut microbiome. Third, alterations in maternal fucosylation may change the distribution and composition of human milk oligosaccharides in breast milk [18, 19], and maternal FUT2 and FUT3 status has been clearly demonstrated to influence the acquisition of natural rotavirus infections in early life [20]. However, existing research exploring associations between infant HBGA phenotype and RVV outcomes has mixed findings [21–23]. Furthermore, few studies have investigated relationships between the combined HBGA status of maternal-infant dyads and RVV underperformance. Therefore, the goal of this study was to characterize the FUT2 and FUT3 phenotype of rural Zimbabwean mothers and their infants and to determine associations with infant RVV immunogenicity.

## METHODS

### Study Population

We conducted a substudy within the Sanitation Hygiene Infant Nutrition Efficacy (SHINE) trial. SHINE was a 2 × 2 factorial cluster-randomized trial investigating the independent and combined effects of a water, sanitation, and hygiene (WASH) intervention and an infant and young child feeding intervention on stunting and anemia (ClinicalTrials.gov NCT01824940). The trial design has been described elsewhere [24]. SHINE enrolled pregnant women who were rural residents of the Chirumanzu and Shurugwi districts in Zimbabwe between 22 November 2012 and 27 March 2015 and their liveborn infants. All women were offered HIV testing and had plasma, urine, stool and saliva stored at −80 °C during pregnancy. From 1 May 2014, a subgroup of infants had plasma, urine, stool, and saliva stored longitudinally between 1 and 18 months of age.

### Rotavirus Vaccination Substudy

The oral monovalent Rotarix vaccine (GSK Biologicals) was introduced into the Zimbabwean Expanded Programme of Immunisation in May 2014. Vaccination at 6 and 10 weeks of age occurred at local clinics, which the trial did not oversee. The national rotavirus vaccination coverage in 2015 to 2016 was between 87% and 91% [25]. Vaccination dates were obtained by trial staff reviewing child health records held by the mother.

For the current analysis, eligibility criteria required infants to be HIV unexposed and live born after the introduction of RVV, to have at least 1 stored plasma sample available before 6 months of age, and to have available stored saliva samples from mother and infant. Participants were excluded from the analysis if infant vaccination records were incomplete or if infants did not receive at least 1 dose of RVV by 6 months of age.

### Laboratory Methods

The main exposures of interest in this analysis were maternal and infant FUT2 and FUT3 phenotypes, which we characterized as secretor (FUT2+) or nonsecretor (FUT2–) status and Lewis (FUT3+) or non-Lewis (FUT3–) status. This was ascertained by the detection of A, B, H, and Lewis a and b antigens in salivary samples via a phenotyping assay, as previously described [26]. Secretors were defined as individuals (mothers or infants) with the presence of A, B, H, or Lewis b antigen, while nonsecretors were defined as those with the presence of Lewis a antigen. Lewis-positive individuals were defined as those with the presence of Lewis a or b antigen, while Lewis-null individuals had neither Lewis a nor Lewis b antigen detected. Those with undetectable A, B, H, Lewis a, and Lewis b were categorized as indeterminate but considered “nonsecretor, Lewis negative” in a sensitivity analysis.

The outcome of interest was infant rotavirus immunogenicity, measured by enzyme-linked immunosorbent assay in plasma, as previously described [27]. The study used anti-rotavirus immunoglobulin A (IgA) seroconversion as the primary outcome, as it is the best current immune correlate of protection against rotavirus infection [28]. Seroconversion was defined as a postvaccination plasma concentration of anti-rotavirus IgA ≥20 U/mL in infants who were seronegative prevaccination (<20 U/mL) [20]. Secondary outcomes were anti-rotavirus seropositivity, defined as postvaccine plasma concentration of anti-rotavirus IgA ≥20 U/mL regardless of prevaccination measurements (binary variable), and anti-rotavirus IgA geometric mean titer (GMT) measured in units per milliliter (continuous variable).

### Statistical Analysis

We used generalized estimating equations with an exchangeable working correlation structure to account for within-cluster correlation [29]. For analysis of the dichotomous outcomes (rotavirus seroconversion and seropositivity), we used a log-binomial specification to facilitate estimation of relative risks; if the log-binomial model did not converge, we used a Poisson model. For the continuous outcome (anti-rotavirus GMT), we employed a log-normal censored regression model (Tobit) with left censoring at 7.5 U/mL to handle values below the assay detection limit. We previously showed an effect of the randomized WASH intervention on RVV immunogenicity [30], and we therefore adjusted for trial arm (WASH/non-WASH) in all models. In addition, we assigned birthweight, length-for-age *Z* score at 3 months, infant age at blood collection, and concurrent oral polio vaccine receipt to the models as covariates based on our previous findings [31]. We included maternal and infant secretor and Lewis status in the same multivariable model. In separate models, we explored the effects of maternal and infant secretor or Lewis status by comparing mother-child pairs who were (1) secretor mother–secretor infant (reference group), secretor mother–nonsecretor infant, nonsecretor mother–secretor infant, and nonsecretor mother–nonsecretor infant and (2) Lewis mother–Lewis infant (reference group), Lewis mother–non-Lewis infant, non-Lewis mother–Lewis infant, and non-Lewis mother–non-Lewis infant.

Since vaccine administration was not undertaken by the trial, we were unable to control receipt and timing of RVV administration in relation to specimen collection. We therefore undertook a sensitivity analysis to restrict timing of titer measurements to 0 to 14 days prevaccination and 7 to 60 days postvaccination in relation to the last dose of Rotarix.

All statistical analyses were performed with Stata version 17 (StataCorp LP).

### Ethics

Ethical approval was provided for the SHINE trial and the current substudy by the Medical Research Council of Zimbabwe and the Johns Hopkins Bloomberg School of Public Health. Written informed consent was obtained from the caregivers of all participating infants before enrollment into the study.

## RESULTS

### Baseline Characteristics

**Figure 1.**
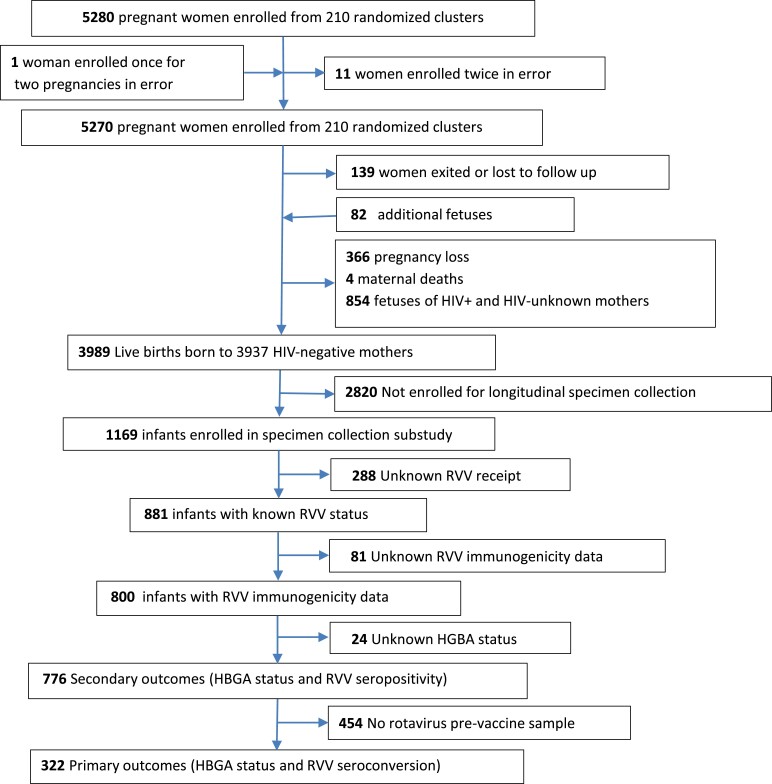
Trial flow. HBGA, histo-blood group antigen; RVV, rotavirus vaccine.

Among 5280 pregnant women recruited to the SHINE trial, there were 3989 liveborn infants who were HIV unexposed (Figure 1). Of these, 776 mother-infant pairs had FUT2 and FUT3 status measured in mothers, infants, or both and available postvaccination anti-rotavirus IgA titers (653 mothers, 707 infants); these mother-infant pairs were included in the analysis of secondary outcomes (anti-rotavirus seropositivity and anti-rotavirus IgA GMT). Among infants, 322 (41.5%) also had prevaccination plasma concentration of anti-rotavirus IgA measured and were included in the primary outcome (seroconversion), which required pre- and postvaccine titers. The characteristics of infants, mothers, and their households are summarized in Table 1. Overall, 554 of 768 (72.1%) mothers and 625 of 776 (80.5%) infants were FUT2 positive, and 492 of 768 (64.1%) mothers and 535 of 776 (68.9%) infants were FUT3 positive.

**Table 1.** Baseline Characteristics of Mothers and Their Infants

Characteristic	Primary Outcome	Secondary Outcome
Infants	322	776
Mothers	319	768
Trial arm		
SOC	104/322 (32.3)	214/776 (27.6)
IYCF	110/322 (34.2)	245/776 (31.6)
WASH	48/322 (14.9)	143/776 (18.4)
ICYF + WASH	60/322 (18.6)	174/776 (22.4)
Household characteristics		
Household size, median (IQR)	5 (3–6)	5 (4–6)
Wealth quintile		
1 (lowest)	49/322 (15.2)	122/776 (15.7)
2 (second)	65/322 (20.2)	157/776 (20.2)
3 (middle)	69/322 (21.4)	161/776 (20.8)
4 (fourth)	74/322 (23.0)	162/776 (20.9)
5 (highest)	56/322 (17.4)	151/776 (19.5)
Unknown	9/322 (2.8)	23/776 (3.0)
Any latrine at household	116/301 (38.5)	308/739 (41.7)
Electricity	8/312 (2.6)	22/752 (2.9)
Maternal characteristics		
Maternal age, y, mean (SD)	26.8 (6.1)	26.9 (6.6)
Parity, median (IQR)	2 (1–3)	2 (1–3)
FUT2 status		
Positive	232/319 (72.7)	554/768 (72.1)
Negative	40/319 (12.5)	99/768 (12.9)
Unknown	47/319 (14.7)	115/768 (15.0)
FUT3 status		
Positive	202/319 (63.3)	492/768 (64.1)
Negative	70/319 (21.9)	161/768 (21.0)
Unknown	47/319 (14.7)	115/768 (15.0)
Infant characteristics		
Female sex	152/322 (47.2)	384/776 (49.5)
Birth weight, kg, mean (SD)	3.14 (0.47)	3.13 (0.46)
Low birthweight	21/312 (6.7)	57/756 (7.5)
Receipt of concurrent oral polio vaccine	273/292 (93.5)	667/709 (94.1)
Exclusive breastfeeding at 3 mo	299/316 (94.6)	672/734 (91.6)
FUT2 status		
Positive	257/322 (79.8)	625/776 (80.5)
Negative	33/322 (10.2)	82/776 (10.6)
Unknown	32/322 (9.9)	69/776 (8.9)
FUT3 status		
Positive	224/322 (69.6)	535/776 (68.9)
Negative	66/322 (20.5)	172/776 (22.2)
Unknown	32/322 (9.9)	69/776 (8.9)

Data are presented as No. (%) unless noted otherwise.

Abbreviations: IYCF, infant and young child feeding; SOC, standard of care; WASH, water, sanitation, and hygiene.

### Primary Outcomes

#### Association Between Maternal FUT2 or FUT3 Status and Vaccine Seroconversion.

Of 234 infants born to women who were FUT2 positive, 47 (20.1%) seroconverted following vaccination, as opposed to 11 (27.5%) of 40 infants born to women who were FUT2 negative (absolute difference, −7.4%; 95% CI, −22.2% to 7.3%). Fully adjusted analyses demonstrated a 53% relative reduction in seroconversion (adjusted relative risk [aRR], 0.47; 95% CI, .26–.82; *P* = .008). Of 204 infants born to women who were FUT3 positive, 37 (18.1%) seroconverted following vaccination, as compared with 21 (30.0%) of 70 infants born to women who were FUT3 negative (absolute difference, −11.9%; 95% CI, −23.8% to .1%). Fully adjusted analyses demonstrated a 55% relative reduction in seroconversion (aRR, 0.45; 95% CI, .25–.78; *P* = .005; Table 2, Supplementary Table 1).

**Table 2.** Associations Between Maternal and Infant FUT2 and FUT3 Status and Infant Rotavirus Vaccine Immunogenicity

	No. (%) or GMT (95% CI)		Difference, RR (95% CI)^a^	*P* Value	Adjusted Difference, aRR (95% CI)^b,c^	*P* Value
	Maternal secretor	Maternal nonsecretor				
Seroconversion	47/234 (20.1)	11/40 (27.5)	0.74 (.43, 1.30)	.300	0.47 (.26, .82)	.008
Seropositivity	130/557 (23.3)	28/100 (28.0)	0.85 (.58, 1.24)	.388	0.73 (.47, 1.14)	.164
IgA, IU/mL	15.8 (14.1, 17.8)	17.0 (12.9, 22.5)	−0.13 (−.49, .24)	.497	−0.14 (−.58, .31)	.549
	Infant secretor	Infant nonsecretor				
Seroconversion	66/257 (25.7)	5/33 (15.2)	1.76 (.71, 4.34)	.220	2.15 (.78, 5.90)	.139
Seropositivity	263/625 (26.1)	14/82 (17.1)	1.54 (.98, 2.42)	.059	1.82 (.98, 3.37)	.057
IgA, IU/mL	17.5 (15.6, 19.8)	11.5 (9.3, 14.2)	0.51 (.00, 1.02)	.050	0.56 (−.01, 1.21)	.055
	Maternal Lewis	Maternal non-Lewis				
Seroconversion	37/204 (18.1)	21/70 (30.0)	0.64 (.41, 1.01)	.058	0.45 (.25, .78)	.005
Seropositivity	102/495 (20.6)	56/162 (34.6)	0.61 (.47, .80)	<.001	0.53 (.38, .75)	<.001
IgA, IU/mL	14.3 (12.7, 16.0)	22.7 (17.5, 29.4)	−0.39 (−.69, −.10)	.009	−0.50 (−.85, −.14)	.006
	Infant Lewis	Infant non-Lewis				
Seroconversion	59/224 (26.3)	12/66 (18.2)	1.42 (.86, 2.34)	.165	1.51 (.80, 2.83)	.202
Seropositivity	142/535 (26.5)	35/172 (20.4)	1.31 (.91, 1.88)	.146	1.65 (1.05, 2.58)	.029
IgA, IU/mL	17.9 (15.7, 20.4)	13.4 (11.1, 16.1)	0.39 (.03, .75)	.032	0.46 (.06, .85)	.023

Abbreviations: aRR, adjusted relative risk; GMT, geometric mean titer; IgA, immunoglobulin A; RR, relative risk.

^a^Difference for IgA: coefficient (95% CI).

^b^Multivariable generalized estimating equation model adjusted for maternal/infant FUT2/FUT3 phenotype, trial arm, birthweight, exact age at time of blood sampling, length-for-age *Z* score, and concurrent receipt of oral polio vaccine.

^c^Difference for IgA: adjusted coefficient (95% CI).

#### Association Between Infant FUT2 or FUT3 Status and Vaccine Seroconversion.

In contrast to maternal phenotypes, infants who were FUT2 or FUT3 positive had a trend toward higher seroconversion than infants who were FUT2 or FUT3 negative, respectively, although these findings were not statistically significant in adjusted models (FUT2 positive [66/257, 25.7%] vs FUT2 negative [5/33, 15.2%]: absolute difference, 10.5%; 95% CI, −2.8% to 23.9%; aRR, 2.15; 95% CI, .78–5.90; *P* = .139; FUT3 positive [59/224, 26.3%] vs FUT3 negative [12/66, 18.2%]: absolute difference, 8.2%; 95% CI, −2.8% to 19.1%; aRR, 1.51; 95% CI, .80–2.83; *P* = .202; Table 2, Supplementary Table 1).

### Secondary Outcomes

#### Association of Maternal FUT2 or FUT3 Status With Vaccine Seropositivity and IgA GMT.

For secondary outcomes, there was no evidence that infants of women who were FUT2 positive had lower seropositivity (aRR, 0.73; 95% CI, .47–1.14; *P* = .164) or GMT (adjusted coefficient −0.14; 95% CI, −.58 to .31; *P* = .549) as compared with infants of women who were FUT2 negative. However, there was strong evidence that infants of women who were FUT3 positive had lower seropositivity (aRR, 0.53; 95% CI, .38–.75; *P* < .001) and GMT (adjusted coefficient −0.50; 95% CI, −.85 to −.14; *P* = .006) than infants of women who were FUT3 negative (Table 2, Supplementary Table 1).

#### Association of Infant FUT2 or FUT3 Status With Vaccine Seropositivity and IgA GMT.

Similar to seroconversion findings, infants who were FUT2 or FUT3 positive had a greater likelihood of being seropositive than infants who were FUT2 or FUT3 negative, respectively (FUT2: aRR, 1.82; 95% CI, .98–3.37; *P* = .057; FUT3: aRR, 1.65; 95% CI, 1.05–2.58; *P* = .029). Correspondingly, FUT2-positive infants had a higher GMT than FUT2-negative infants (adjusted coefficient, 0.56; 95% CI, −.01 to 1.21; *P* = .055), and FUT3-positive infants had a higher GMT than FUT3-negative infants (adjusted coefficient, 0.46; 95% CI, .06–.85; *P* = .023; Table 2, Supplementary Table 1).

#### Combined Mother-Infant Dyad Phenotypes.

Finally, we evaluated infant rotavirus seroconversion according to the combined mother-infant HBGA phenotype. When compared with a reference category of FUT2-positive infants with FUT2-positive mothers (the most common combined phenotype in the study population), FUT2-positive infants with FUT2-negative mothers were twice as likely to seroconvert (7/19 [36.8%] vs 43/196 [21.9%]; aRR, 2.08; 95% CI, 1.19–3.65; *P* = .011). Among FUT2-negative infants with FUT2-positive mothers, there were no seroconverters (0%). When compared with FUT3-positive infants with FUT3-positive mothers, FUT3-positive infants with FUT3-negative mothers were almost 3 times more likely to seroconvert (14/29 [48.3%] vs 29/159 [18.2%]; aRR, 2.81; 95% CI, 1.64–4.80; *P* < .001; Table 3).

**Table 3.** Seroconversion by Combined Maternal and Infant Secretor and Lewis Status

	Maternal Secretor (FUT2+)	*P* Value	Maternal Nonsecretor (FUT2−)	*P* Value
Infant secretor (FUT2+)				
No. (%)	43/196 (21.9)		7/19 (36.8)	
RR (95% CI)	Reference		1.63 (.94–2.82)	.079
aRR^a^ (95% CI)	…		2.08 (1.19–3.65)	.011
Infant nonsecretor (FUT2−)				
No. (%)	0/14 (0)		3/13 (23.1)	
RR (95% CI)	…		1.03 (.36–2.89)	.962
aRR^a^ (95% CI)	…		1.70 (.58–5.00)	.331

	Maternal Lewis (FUT3+)		Maternal Non-Lewis (FUT3−)	
Infant Lewis (FUT3+)				
No. (%)	29/159 (18.2)		14/29 (48.3)	
RR (95% CI)	Reference		2.56 (1.59–4.12)	<.001
aRR^a^ (95% CI)	…		2.81 (1.64–4.80)	<.001
Infant non-Lewis (FUT3−)				
No. (%)	5/21 (23.8)		5/33 (15.2)	
RR (95% CI)	1.44 (.68–3.06)	.342	0.80 (.34–1.87)	.603
aRR^a^ (95% CI)	1.53 (.76–3.10)	.235	0.91 (.37–2.20)	.831

Abbreviations: aRR, adjusted relative risk; RR, relative risk.

^a^Adjusted for maternal/infant FUT2/FUT3 phenotype, trial arm, birthweight, exact age at time of blood sampling, and length-for-age *Z* score. Models including concurrent oral polio vaccine receipt did not converge due to insufficient number of infants who did not receive oral polio vaccine (≤5 in each comparison group).

### Sensitivity Analyses

When mothers and infants with undetectable A, B, H, Lewis a, and Lewis b antigens were considered “nonsecretor, Lewis negative,” there was no difference in inference (Supplementary Table 2). When the timing of pre- and postvaccine titer measurements (in relation to the last dose of Rotarix) were restricted to a tighter window, there were no changes in the inferences of any findings, although the precision of the estimates was reduced (data not shown).

## DISCUSSION

This study set out to characterize FUT2 and FUT3 phenotypes in rural Zimbabwean mother-infant pairs and to determine associations with infant RVV immunogenicity. Specifically, the study aimed to explore whether genetic factors that determine HBGA status contribute to the impaired efficacy of oral vaccines in low-income countries. There are 3 main findings. First, FUT2- and FUT3-positive phenotypes predominated in this study population. Second, mothers’ FUT2- and FUT3-positive status was strongly associated with reduced rotavirus immunogenicity in their infants. Third, in FUT2- or FUT3-negative women, infants who were FUT2 or FUT3 positive, respectively, had improved rotavirus immunogenicity. Taken together, these data highlight (1) the potential role of HBGA phenotypes in shaping infant vaccine responses and (2) the particular influence that maternal FUT2- and FUT3-positive phenotypes may have in reducing infant RVV immunogenicity in a setting where the majority of women are Lewis-positive secretors.

Few studies have investigated associations between maternal HBGA phenotypes and infant RVV seroconversion, and findings have been mixed [21–23]. This heterogeneity may be related to the influence of geographic variation in phenotypes. For example, it has been reported that FUT2-negative phenotypes are more common in people from parts of Bangladesh, India, and Pakistan, with a prevalence of 30% to 50%, as opposed to 20% in Caucasians [32–34], and FUT3-negative phenotypes are more prevalent in certain parts of Africa (>30%) as compared with Caucasian populations (4%–6%) [35]. Our analysis of mother-infant dyads suggests that maternal phenotype has a greater influence on RVV immunogenicity than infant phenotype. We found strong evidence that infants with a FUT2- or FUT3-positive mother had >50% reduction in seroconversion. These findings could be explained by the presence of breast milk components that directly inhibit the interaction of vaccine strains with small intestinal epithelial cells [36]. It has been shown in vitro that 2-fucosyllactose—1 of the 3 major human milk oligosaccharide types found in the breast milk of FUT2-positive mothers—can reduce the infectivity of the 2 most globally prevalent strains of rotavirus, P[8] and P[4] [37]. HBGA phenotype-dependent glycans in breast milk can act as decoy receptors for RVV [38], meaning that RVV binds less efficiently to receptors on the gut epithelium, which prevents uptake of the vaccine. However, the effects of breastfeeding on oral vaccine immunogenicity are heterogeneous [39, 40], and some studies have reported higher oral polio vaccine immunogenicity in breastfed vs nonbreastfed infants [41, 42]. Also, in a study that measured the association of rotavirus risk among infants in early life and FUT2/FUT3 status among mother-child dyads, there was a higher risk of rotavirus detection in children of FUT3-positive mothers for combined rotavirus serotypes and P[8] infections, though not P[6] ones [20], suggesting that perhaps impaired immune responses lead to more natural infections. Alternatively, maternal antibody responses to rotavirus may have differed by maternal HBGA phenotype with subsequent modification of infant vaccine responses.

Contrary to our hypothesis, we did not find strong evidence of associations between infant HBGA status and seroconversion to RVV, although the point estimates of the relative risk of seroconversion suggested that FUT2- or FUT3-positive infants were more likely to seroconvert than FUT2- or FUT3-negative infants. In secondary outcomes, there was evidence of higher seropositivity and GMT among infants who possessed FUT2 or FUT3. Our analysis of mother-infant dyads points to an interplay between maternal and infant HBGA status: FUT2- or FUT3-positive infants who had FUT2- or FUT3-negative mothers were 2 to 3 times more likely to seroconvert following vaccination. It was striking that among maternal secretor and infant nonsecretor pairs, none of the infants seroconverted following vaccination (Table 3). There is some evidence that HBGAs are used as attachment factors by enteric pathogens and pathogen-derived toxins, resulting in HBGA-dependent susceptibility [9]. Furthermore, such interactions may occur in a genotype-specific manner, with 1 study reporting a 27-fold increase in susceptibility to P[8] rotavirus in secretors vs nonsecretors [14]. Since P[8] is a component of the Rotarix vaccine administered in our study population, this could be one explanation for greater immunogenicity in FUT2- or FUT3-positive infants, particularly when the interfering effects of maternal phenotype are absent. Taken together, the combination of maternal and infant HBGA phenotype may critically shape responses to vaccination, highlighting the importance of considering the mother-infant pair in tandem.

There are strengths and limitations to this study. Mother-infant pairs were drawn from a large birth cohort in rural Zimbabwe, with proven poor seroconversion to oral vaccines. This was a well-characterized study population with rich covariate data allowing for multivariable analyses and samples available for HBGA phenotyping. However, this was a substudy that included only mother-infant pairs with sufficient sample volume and infants with existing rotavirus immunogenicity data, meaning that the cohort may not be representative of the whole trial population. We did not measure maternal rotavirus responses, which could have differed according to maternal HBGA phenotype and thus influenced infant vaccine responses. We did not assess natural infant rotavirus infection, which may also have influenced IgA titers. The combination of low seroconversion and a relatively small sample size increased the risk of a type II error. Furthermore, as the administration of the vaccine was not part of the trial, the timing of the pre- and postvaccine blood sample collection was not controlled; although we addressed this with a prespecified sensitivity analysis, it would be valuable to acquire further data from specific vaccination studies with fixed timing of pre- and postvaccination blood sampling.

In summary, this study provides insights into the associations between HBGA phenotype and infant RVV immunogenicity in a setting with very poor seroconversion rates. We show that FUT2- and FUT3-positive maternal phenotypes, which predominate in this population, may be one factor explaining the poor immunogenicity of RVV in Zimbabwean infants. By contrast, infants with positive secretor or Lewis status had some evidence of better oral vaccine immunogenicity, particularly if their mothers were FUT2 or FUT3 negative. Overall, our results highlight the impact of maternal and infant HBGA phenotypes on infant rotavirus immunogenicity. This work contributes to our growing understanding of the mechanisms underlying poor RVV immunogenicity in low-income countries, which is essential to inform future interventions.

## Supplementary Data

Supplementary materials are available at *The Journal of Infectious Diseases* online (http://jid.oxfordjournals.org/). Supplementary materials consist of data provided by the author that are published to benefit the reader. The posted materials are not copyedited. The contents of all supplementary data are the sole responsibility of the authors. Questions or messages regarding errors should be addressed to the author.

## Supplementary Material

jiae456_Supplementary_Data

## Notes

***Author contributions*.** The study was conceived by J. A. C., J. H. H., B. D. K., M. K., R. N., and A. J. P. Participant recruitment and data and sample collection were overseen by F. D. M., N. V. T., J. H. H., and A. J. P. Laboratory work was undertaken by J. A. C., K. M., S. R., M. G., and P. M. Analysis was undertaken by J. P., B. C., E. G., C. E., and R. N. Interpretation of findings was undertaken by J. P., B. C., J. A. C., E. G., C. E., R. N., and A. J. P. Study funding and oversight were from J. A. C., J. H. H., M. K., and A. J. P. The manuscript was drafted by J. P., C. E., and A. J. P., with critical input and revision from all authors.

***Financial support*.** This work was supported by the Wellcome Trust (203905/Z/16/Z to J. A. C., 093768/Z/10/Z and 108065/Z/15/Z to A. J. P., and 203905/Z/16/Z and 210807/Z/18/Z to C. E.). The SHINE trial was funded by the Bill and Melinda Gates Foundation (OPP1021542 and OPP113707); UK Department for International Development (UK Aid); Swiss Agency for Development and Cooperation; US National Institutes of Health (2R01HD060338-06); and UNICEF (PCA-2017-0002). C. E. is funded by the National Institute for Health and Care Research.
