# Supplementary material for: Associations Between Histo-blood Group Antigen Status in Mother-Infant Dyads and Infant Oral Rotavirus Vaccine Immunogenicity in Rural Zimbabwe
Source: J Infect Dis. 2024 Oct 1;231(1):e225–33. doi: 10.1093/infdis/jiae456 (PMC11793023; doi:10.1093/infdis/jiae456)
Supplement: jiae456_Supplementary_Data [file jiae456_supplementary_data.docx]

|  | **Maternal/infant secretor/Lewis** | | **Absolute difference** | ***P*** |
| --- | --- | --- | --- | --- |
| Seroconversion | **Maternal secretor**  47/234 (20.1%) | **Maternal non-secretor**  11/40 (27.5%) | -7.41 (-22.22, 7.34) | 0.29 |
| Seropositivity | 130/557 (23.3%) | 28/100 (28.0%) | -4.66 (-14.14, 4.81) | 0.32 |
|  | **Infant secretor** | **Infant non-secretor** |  |  |
| Seroconversion | 66/257 (25.7%) | 5/33 (15.2%) | 10.53 (-2.82, 23.88) | 0.19 |
| Seropositivity | 263/625 (26.1%) | 14/82 (17.1%) | 9.01 (0.17, 17.85) | 0.08 |
|  | **Maternal Lewis** | **Maternal non-Lewis** |  |  |
| Seroconversion | 37/204 (18.1%) | 21/70 (30.0%) | -11.86 (-23.83, 0.10) | 0.04 |
| Seropositivity | 102/495 (20.6%) | 56/162 (34.6%) | -13.96 (-22.11, -5.82) | <0.001 |
|  | **Infant Lewis** | **Infant non-Lewis** |  |  |
| Seroconversion | 59/224 (26.3%) | 12/66 (18.2%) | 8.16 (-2.79, 19.11) | 0.18 |
| Seropositivity | 142/535 (26.5%) | 35/172 (20.4%) | 6.19 (-0.89, 13.28) | 0.10 |

**Supplementary Table 1. Absolute differences in rotavirus seroconversion and seropositivity by maternal and infant FUT2 and FUT3 status.**

|  |  |  | Adjusted difference* | *P* |
| --- | --- | --- | --- | --- |
| Seroconversion | **Maternal secretor**  47/234 (20.1%) | **Maternal non-secretor****  24/75 (32.0%) | aRR 0.65 (0.43, 0.99) | 0.045 |
| Seropositivity | 130/557 (23.3%) | 52/176 (29.6%) | aRR 0.82 (0.60, 1.10) | 0.182 |
| IgA GMT (95%CI), IU/mL | 15.8 (14.1, 17.8) | 18.4 (14.8, 22.9) | Adjusted co-efficient -0.06 (-0.18, 0.06) | 0.322 |
|  | **Infant secretor** | **Infant non-secretor**** |  |  |
| Seroconversion | 66/257 (25.7%) | 10/71 (14.1%) | aRR 1.55 (0.79, 3.05) | 0.205 |
| Seropositivity | 163/625 (26.1%) | 32/167 (19.2%) | aRR 1.26 (0.85, 1.89) | 0.249 |
| IgA GMT (95%CI), IU/mL | 17.5 (15.6, 19.8) | 12.3 (10.5, 14.4) | Adjusted co-efficient 0.10 (-0.03, 0.23) | 0.142 |
|  | **Maternal Lewis** | **Maternal non-Lewis**** |  |  |
| Seroconversion | 37/204 (18.1%) | 34/105 (32.4%) | aRR 0.54 (0.34, 0.86) | 0.009 |
| Seropositivity | 102/495 (20.6%) | 80/238 (33.6%) | aRR 0.61 (0.46, 0.80) | <0.001 |
| IgA GMT (95%CI), IU/mL | 14.3 (12.7, 16.0) | 22.0 (17.9, 27.0) | Adjusted co-efficient -0.22 (-0.33, -0.11) | <0.001 |
|  | **Infant Lewis** | **Infant non-Lewis**** |  |  |
| Seroconversion | 59/224 (26.3%) | 17/104 (16.4%) | aRR 1.39 (0.84, 2.34) | 0.216 |
| Seropositivity | 142/535 (26.5%) | 53/257 (20.6%) | aRR 1.41 (1.02, 1.95) | 0.037 |
| IgA GMT (95%CI), IU/mL | 17.9 (15.7, 20.4) | 13.3(11.5, 15.4) | Adjusted co-efficient 0.17 (0.06, 0.29) | 0.003 |

**Supplementary Table 2. Sensitivity analysis for associations between maternal and infant FUT2 and FUT3 status and infant rotavirus vaccine immunogenicity using alternate categorisation**

*Multivariable generalised estimating equation model adjusted for maternal/infant FUT2/FUT3 phenotype, trial arm, birthweight, exact age at time of blood sampling, length-for-age Z-score, and concurrent receipt of oral polio vaccine. RR: relative risk; aRR: adjusted relative risk; GMT: Geometric mean titre.

**Alternate categorisation: mothers and infants with undetectable A, B, H, Lewis a and Lewis b categorised as non-secretor, Lewis-negative.
